# Targeting Inflammation in Emerging Therapies for Genetic Retinal Disease

**DOI:** 10.1155/2013/581751

**Published:** 2013-02-21

**Authors:** Ishaq A. Viringipurampeer, Abu E. Bashar, Cheryl Y. Gregory-Evans, Orson L. Moritz, Kevin Gregory-Evans

**Affiliations:** Eye Care Centre, Department of Ophthalmology and Visual Science, University of British Columbia, 2550 Willow Street, Vancouver, BC, Canada V5Z 3N9

## Abstract

Genetic retinal diseases such as age-related macular degeneration and monogenic diseases such as retinitis pigmentosa account for some of the commonest causes of blindness in the developed world. Diverse genetic abnormalities and environmental causes have been implicated in triggering multiple pathological mechanisms such as oxidative stress, lipofuscin deposits, neovascularisation, and programmed cell death. In recent years, inflammation has also been highlighted although whether inflammatory mediators play a central role in pathogenesis or a more minor secondary role has yet to be established. Despite this, numerous interventional studies, particularly targeting the complement system, are underway with the promise of novel therapeutic strategies for these important blinding conditions.

## 1. Introduction

Inherited retinal diseases include some of the commonest causes of blindness in the developed world [1, 2]. Prominent examples included age-related macular degeneration (AMD), diabetic retinopathy, and the numerous monogenic conditions such as retinitis pigmentosa (RP), Stargardt's disease, and X-linked retinoschisis. As well as causative/predisposing genetic abnormalities [3], in some cases (such as AMD), environmental factors such as smoking and diet have been highlighted [4, 5]. These aetiological factors have been associated with diverse abnormal biochemical pathways in the degenerating retina, for instance, oxidative stress [6]; lipofuscin accumulation in the retinal pigment epithelium [7]; abnormalities of the extracellular matrix [8]; mitochondrial abnormalities [9]; ischaemia with neovascularisation [10]; programmed cell death [11]. In particular in AMD and RP, inflammation has recently become a prominent member of this list of abnormal pathological pathways triggered by genetic retinal disease [12, 13]. 

## 2. Established Links between Inflammation and Genetic Retinal Disease

Early studies showed that autoantibodies can be detected in blood of AMD patients [14, 15] and that macrophages also accumulate in the choroid [16] which suggested that immune-mediated processes were involved in the pathogenesis of AMD. Renewed interest in inflammation in genetic retinal disease was, however, more recently triggered by the discovery of elements of the immune system and multiple proteins of the complement pathway within the drusen seen in AMD [17]. Although the exact pathophysiology of AMD remains largely unknown, accumulation of drusen is acknowledged as an early and major pathological hallmark of the disease, preempting damage in the retinal pigment epithelium, photoreceptors, and choroid leading to atrophic or neovascular complications. 

Immunohistochemical studies of human retina highlighted that amongst other components, AMD drusen contained inflammatory mediators such as vitronectin, immunoglobulin light chains, factor X, and complement proteins (C5 and C5b-9 complex). Importantly, it was also demonstrated that drusen displays intense HLA-DR immunoreactivity [17, 18]. This later finding complements other independent work suggesting and association between AMD and HLA-genotype [19]. It is, however, now recognised that the composition of drusen deposits is extremely heterogeneous amongst different patients and that many other components are also found in addition to inflammatory mediators. For instance, other studies have highlighted the appearance of oxidative protein modifications within AMD drusen including cross-linked species of tissue metalloproteinase inhibitor 3, vitronectin, and carboxyethyl pyrrole protein adducts suggesting oxidative stress as an etiological factor in AMD drusen formation [20].

The feasibility of linking immunity to AMD pathophysiology has also been suggested by the central role the retinal pigment epithelium (RPE) plays in both AMD pathogenesis [21] and ocular immune modulation [22]. Both *in vivo *and* in vitro* experiments have demonstrated that the RPE expresses both innate and adaptive immune receptors [22, 23]. In addition, RPE cells are known to secrete numerous cytokines, chemokines, and adhesive molecules including interleukin-6, interleukin-8, and immunosuppressive factors including tissue-necrosis factor-*β*, interleukin-11, and interferon-*β* [22]. 

Evidence suggesting a role for inflammation in AMD has, however, been strongly supported by molecular genetic studies. In particular, genes encoding components of the complement pathways have been associated with AMD. Strong associations have been demonstrated for alleles of genes encoding complement factor H (*CFH*) [24–26] which is a regulator of the alternate complement pathway, complement component C2/complement factor B (*C2/CFB*) [27], and CFH-related genes* CFHR1* and *CFHR3* [28]. Weaker associations have been linked to complement factor 1; complement C3 and complement component 1 inhibitor (*SERPING1*), a regulator of the classic complement pathway; chemokine C-X3-C receptor 1 (*CX3Cr1*); and toll-like receptor genes *TLR3* and *TLR4* (with a role in the innate immune system) [29, 30]. It should be noted, however, that strong genetic associations between loci and AMD also exist, for example, to the *PLEKHA1/ARMS2/HTRA1* region of chromosome 10q26 [29].

Most recently, however, there has been remarkable work linking nucleotide-binding domain and leucine-rich-repeat-protein 3 (NLRP3) and the “inflammasome” with the aetiology of ARMD [31–33]. The inflammasome is a term used to identify a collection of proteins that work together within cells with a common purpose, more specifically, a caspase-1 dependant multiprotein complex that has a key role in innate immunity. The inflammasome can be triggered by a number of stimuli including microbial pathogen-associated molecular patterns, bacterial toxins and most relevant to genetic retinal disease “damage-associated molecular patterns” (denatured nuclear or cytosolic proteins released from dying cells). This results in the upregulation of proinflammatory cytokines interleukin-1*β* and interleukin-18 [34, 35]. The inflammasome can be activated by three classes of immune sensors including the toll-like receptors, RIG-I-like helicases, and NLR proteins [36]. Currently, four inflammasomes based on NLRP3 have been characterised: NLRP1/NLRP1b [37]; NLRC4/IPAF [38]; NLRP3/NALP3 [39]; AIM2 [40]. Activation of the NLRP3 inflammasome has recently been reported in dry AMD drusen, by complement component C1Q or by carboxyethylpyrrole (during oxidative stress) [31]. In wet ARMD, activation of the NLRP3 inflammasome has been seen triggered by Alu RNA molecules (short chains RNA) [32]. Intriguingly, reflecting the diversity of immune responses, the most recent *in vivo *and *in vitro* studies have suggested that the NLRP3 inflammasomes may have a beneficial role in wet ARMD but a harmful effect leading to RPE cell death in dry AMD [31, 32].

Linking inflammatory mediators to the choroidal neovascularization seen in complicated AMD is also firmly established. In both preclinical and clinical studies, cellular components of immunity including macrophages, lymphocytes and neutrophils have been found to be significant components of choroidal neovascular complexes [16, 41, 42]. Additionally, inflammatory cytokines such as interleukin-6 and interleukin-8 have also been identified in the aqueous humor of AMD patients suffering from choroidal neovascularization [43]. 

While the term “retinitis pigmentosa” as coined by Donders in 1857 is generally considered a misnomer, a role for inflammation and immunity in the pathogenesis of the disease has significant merit. The earliest studies to suggest this showed elevated IgM in six out of ten RP patients [44]. Other early studies also suggested that retinal (probably photoreceptor) autoantibodies could be found in the systemic circulation of RP patients [45–48]. Immune reactivity in RP was also established by exposing lymphoctyes and leuckocytes from blood samples of RP patients to human and bovine retinal antigens [49]. However, results have been complicated by the fact that immune reactivity appears to vary amongst RP patients possibly reflecting the genetic heterogeneity of the disease. Studies suggested that circulating immune complexes could be detected in less than 50% of RP patients [50]. This was, however, reported as significant since it correlated with statistically significant reductions of circulating complement factors C3 and C4 and a significant reduction in time taken for RP patient sera to achieve 50% hemolysis of sheep red blood cells [50]. A link with HLA status has also been reported in RP patients [51], and vitreous samples from RP patients have been shown to contain many immune system cells such as various types of lymphocytes [52].

More recent studies have highlighted the activation of microglia in RP retina preceding photoreceptor cell death. Activation of microglia results in many biochemical events including the release of cytokines and chemokines [53]. In *rd* mice (a homozygous nonsense phosphodiesterase-beta subunit gene mutant), it has been shown that prior to peak photoreceptor cell death, there is an upregulation of mRNA of proinflammatory factors: monocyte chemoattractant proteins 1 and 3; macrophage inflammatory proteins 1alpha and 1beta; regulated on activation normal T-cell expressed and secreted (RANTES); tumor necrosis factor-alpha [54]. Microglia activation and upregulation of proinflammatory markers has also been demonstrated to precede peak photoreceptor cell death in *rd10* mice (homozygous missense phosphodiesterase beta subunit mutant) [55].

Although direct biochemical assessment of retina from human RP patients is difficult, more detailed recent studies have searched for signs of inflammatory cells and humoral inflammatory factors in aqueous and vitreous humor from RP patients [13]. Slit lamp examination revealed that cells could be visualised in the anterior chamber 37% (190) and the vitreous of 61% (313) of 509 RP eyes (up to 30 cells identified in a 1 × 9 mm vertical slit-lamp field). It was not explained however how it was concluded that these were inflammatory cells (and not for instance, pigmentary cells). The study did, however, also report multiplex ELISA data suggesting significantly increased protein levels of cytokines (interleukin 6) and chemokines (interleukin-8, monocyte chemoattractant protein 1, and thymus activation-regulated chemokine) in the aqueous. Much more notably, in the vitreous of these RP patients, significantly elevated levels of cytokines (interleukin 1*α*, 1*β*, 2, 4, 6, and 10, interferon-*γ* and tissue necrosis factor-*α*) along with chemokines (such as interleukin-8, monocyte chemoattractant proteins 1 and 2, interferon-*γ* inducible protein-10, and thymus activation-regulated chemokine) were found [13].

## 3. Therapeutic Targets

Numerous therapeutic strategies are emerging in the treatment of genetic retinal disease. These include cell-based therapies [56]; gene therapy [57]; electronic retinal replacements [58]; molecular-based approaches such as neuroprotection [59] and antiangiogenesis [60]. With an established role for inflammatory mediators in the pathogenesis of both AMD and RP, it would therefore be rational to investigate anti-inflammatory approaches. However, despite numerous animal models for RP [61], no universally recognised animal model for AMD yet exists, and only approximations modeling aspects of the disease are available [62]. This has limited preclinical research into AMD treatment. In addition, it is as yet unclear what role relative to other pathogenic mechanisms inflammation plays in disease pathogenesis. Anti-inflammatory approaches, for example, might have little impact on disease if inflammation is a secondary effect or a minor contributor to pathogenesis. To some extent, therefore, the importance of inflammation in AMD and RP can be validated through quantification of the effect of anti-inflammatory therapeutics.

There are currently 846 listed clinical trials (http://www.clinicaltrials.gov/) focused on AMD of which 51 are specifically targeting inflammation. There are also 78 clinical trials listed for RP, although none are focused on anti-inflammatory therapeutics. Studies targeting inflammation in AMD and RP may be subdivided into broad approaches targeting multiple components of inflammation or more specific targeting of complement activation, for instance. 

### 3.1. Broad-Based Anti-Inflammatory Studies in AMD

Corticosteroids have been used in retinal disease for many years because of their general anti-inflammatory effect, generally up-regulating the expression of anti-inflammatory proteins and suppressing the expression of proinflammatory factors [63, 64]. Although their use in uveitis and retinitis is well established, corticosteroids are now being considered in AMD. Many studies have looked at the use of intravitreal dexamethasone in the treatment of neovascular complications of AMD, mostly as an adjuvant therapy in combination with photodynamic therapy, intravitreal anti-VEGF therapy, or in multitherapy approaches [65, 66]. A sustained release dexamethasone (such as Ozurdex, Allergan, Inc., Irvine, CA) would seem to be the logical option in such an adjuvant approach. One single-masked, randomized control study in 243 eyes reported work comparing intravitreal ranibizumab plus sustained release dexamethasone with ranibizumab plus sham. Results suggested a reduction in the need for multiple ranibizumab injections and an increasing interval between injections although combination therapy was associated with raised intraocular pressure requiring treatment in 16% [67, 68]. A number of other clinical trials are now assessing dexamethasone as an adjunct in the treatment of wet AMD, and full reports are awaited (http://www.clinicaltrials.gov/, NCT01162746; NCT00793923; NCT00390208).

Other approaches include Iluvien, an intravitreal implant containing fluocinolone acetonide packed in a nonbiodegradable polyamide tube [69]. An ongoing phase 2 clinical trial is currently recruiting patients to look at the efficacy of this intravitreal implant in inhibiting geographic atrophy progression in AMD (http://www.clinicaltrials.gov/, NCT00695318).

Rapamycin, a drug originally designed as an antifungal agent, has recently been shown to be a potent immunosuppressive, anti-inflammatory, and antiangiogenesis agent by inhibiting the mammalian target of rapamycin (mTOR), a serine/threonine protein kinase. Recent work in the senescence-accelerated OXYS rat has shown some inhibition of the spontaneous retinopathy phenotype seen which models age-related macular degeneration in some respects [70]. A phase 2 study has been launched by National Eye Institute to determine whether repeated intravitreal rapamycin can slow progression of geographic atrophy (http://www.clinicaltrials.gov/, NCT01445548). Glatiramer acetate is another broad immunomodulatory agent that upregulates specific suppressor T-cells and suppresses inflammatory cytokines. Intravenous glatiramer acetate in dry AMD patients has been shown to reduce drusen load on optical coherence tomography imaging [71]. In the “wet” form of AMD, anti-inflammatory agents have been found to be moderately successful in controlling choroidal neovascularization. For example, intravitreal triamcinolone acetonide and infliximab, an antibody of tumor necrosis factor *α* (TNF-*α*), have shown positive effects in treating CNV in patients and animal models [72, 73].

### 3.2. Focused Targeting of the Complement System in AMD

The area in which inflammatory mediators in genetic retinal disease is being most intensively investigated, however, is in inhibiting activation of the complement system in AMD patients [74]. These studies have focused on both antibody dependent and independent complement inhibition trials. 

FCFD4514S is a recombinant, humanized monoclonal antibody Fab fragment antibody targeted to block the complement factor D, an early rate-limiting enzyme in the activation of the alternative complement pathway. Current phase 2 clinical trials are assessing its use in geographic atrophy (http://www.clinicaltrials.gov/, NCT01602120). Eculizumab (Soliris) is a humanized complement factor 5 monoclonal antibody that binds to complement factor 5 to block subsequent downstream anaphylatoxin activation and the formation of membrane attack complexes. It is considered by some to be the most likely approach since it preserves production of C3a anaphylatoxin and C3b production required for opsonisation and clearing of harmful immune complexes [64]. Intravenous eculizumab is currently under trial for dry AMD (http://www.clinicaltrials.gov/, NCT00935883).

An example of studies focusing on nonantibody approaches to modify complement activation is POT-4, the first complement inhibitor to be assessed in AMD. This molecule is a cyclic peptide that reversibly binds to C3 preventing its activation to C3a and C3b, thus blocking the classic pathway and the lecithin pathway as well as the alternative complement pathway. Intravitreal depot injections are being assessed in patients with wet and dry AMD (http://www.clinicaltrials.gov/, NCT00473928).

Other nonantibody approaches include small molecule peptidomimetic C5a receptor antagonists currently being considered for AMD [75]. An alternative approach that may avoid the general consequences of inhibiting complement activation is studies focusing on replacing abnormal complement factor H alleles. TT30 is a recombinant fusion protein being used to replace defective complement factor H [76] and is being considered for use in AMD trials. Modification of complement activation may also be of benefit in wet AMD. In a laser-induced mouse model of choroidal neovascularization, intravenous administration of “CR2-fH,” a recombinant form of complement factor H linked to complement receptor 2, inhibited neovascular growth [77].

### 3.3. Broad-Based Anti-Inflammatory Studies in RP

Corticosteroids have been routinely used to treat the macular edema seen in late RP with mixed success [78]. Most recently, however, a sustained-release dexamethasone implant (Ozurdex) has been used in a small cohort of RP patients with macular oedema, suggesting some structural and functional benefits [79]. Also recently, *N*-acetylcysteine, an orally bioavailable antioxidant, has been used in *rd10* mice [55]. It was shown by TUNEL staining that a reduction in photoreceptor cell death was associated with a strong suppression of expression of cytokines interleukin 1*β* and tissue necrosis factor-*α* and chemokines monocyte chemoattractant proteins 1 and thymus activation-regulated chemokine [55]. In another study, fluocinolone acetonide has been conjugated with dendrimer particles (a hydroxyl-terminated polyamidoamine dendrimer-drug conjugate nanodevice) to target outer retina activated microglia [80]. They showed that after intravitreal administration in the Royal College of Surgeons rat model of RP (homozygous *Mertk* mutant), four weeks later, there had been significant preservation of outer nuclear layer thickness (indicative of photoreceptor survival) and in electroretinogram b-wave response. In addition, it was shown that this was associated with a reduction of activated microglia in the retina [80]. 

## 4. Conclusion 

Considerable evidence now exists linking inflammatory mediators to genetic retinal diseases such as AMD and RP. It is, however, still unclear whether this is a central or pivotal role [81]. Preclinical and clinical trials suggest that inhibiting inflammatory mediators can have some therapeutic benefit, but further ongoing trials are needed to demonstrate the true impact of this approach. The benefits of inhibiting inflammation in genetic retinal disease might, for instance not be so clear-cut. In recent clinical trials, ciliary-derived neurotrophic factor (CNTF, a neuroprotective growth factor) has been shown to provide some inhibition of degeneration in both dry AMD [82] and RP [83]. Studies in mouse retina, however, have suggested that CNTF induces expression of proinflammatory genes in retinal Müller cells [84]. This would seem counterintuitive, and its clinical relevance has yet to be determined. A role for anti-inflammatory agents, as stand-alone monotherapies or as adjuvants (for instance, in combination with neuroprotective strategies or anti-VEGF therapies), is certain to be prominent feature of future research into the treatment of retinal disease.
